# lncRNA NEAT1 Downregulation Ameliorates the Myocardial Infarction of Mice by Regulating the miR-582-5p/F2RL2 Axis

**DOI:** 10.1155/2022/4481360

**Published:** 2022-12-03

**Authors:** Zhenhua Wu, Yunpeng Bai, Yujuan Qi, Chao Chang, Yan Jiao, Yaobang Bai, Zhigang Guo

**Affiliations:** ^1^Academy of Medical Engineering and Translational Medicine, Tianjin University, China; ^2^ICU, Department of Cardiac Surgery, Tianjin Chest Hospital, China; ^3^Department of Cardiac Surgery, Tianjin Chest Hospital, China

## Abstract

**Background:**

This study is aimed at effectively investigating the role of coagulation factor II thrombin receptor like 2 (F2RL2) in myocardial infarction (MI) as well as the upstream regulatory miRNA and lncRNA.

**Methods:**

Regulatory genes of F2RL2 were analyzed using StarBase and verified by dual-luciferase reporter assay. The MI mouse model was established. The left ventricular ejection fraction (EF) and fractional shortening (FS) were examined by echocardiography. The infarct area, pathological changes, and cell apoptosis in mouse myocardial tissue were evaluated using triphenyltetrazolium chloride and Evans blue, hematoxylin-eosin, and TUNEL staining assays. Oxygen-glucose deprivation- (OGD-) induced human cardiac myocytes (HCMs) were cultured and transfected. The cell viability, proliferation, and apoptosis were determined by CCK-8, EdU staining, and flow cytometry assays. The expressions of F2RL2, miR-582-5p, and nuclear paraspeckle assembly transcript 1 (NEAT1) in myocardial tissues and HCMs were quantified by qRT-PCR or Western blot.

**Results:**

NEAT1 sponged miR-582-5p which targeted F2RL2. NEAT1 and F2RL2 were highly expressed while miR-582-5p was lowly expressed in MI mice. F2RL2 downregulation prevented the reduction in EF and SF and the elevation in infarct area and cell apoptosis of MI mice. Both F2RL2 and NEAT1 downregulations reversely modulated the decreased viability and proliferation and the increased apoptosis of OGD-induced HCMs, while miR-582-5p inhibitor did oppositely. NEAT1 silencing upregulated miR-582-5p level but downregulated F2RL2 level. miR-582-5p inhibitor upregulated the F2RL2 level. The role of NEAT1 silencing in OGD-induced HCMs was reversed by miR-582-5p inhibitor whose effect was further offset by F2RL2 downregulation.

**Conclusion:**

NEAT1 downregulation ameliorates MI by regulating the miR-582-5p/F2RL2 axis, providing novel biomarkers for MI treatment.

## 1. Introduction

Acute myocardial infarction (MI) is an extremely severe cardiovascular disease worldwide with the highest incidence rate and mortality rate, which is characterized by myocardial necrosis resulting from acute or persistent ischemia and hypoxia in the myocardial tissue [1–3]. In the context of improved medical conditions, the mortality rate of MI is still not optimistic. Therefore, it is necessary to find out biomarkers with broad application prospects in improving diagnosis, treatment, and prognosis of MI.

Coagulation factor II thrombin receptor like 2 (F2RL2), a G-protein-coupled receptor (GPCR) which encodes protease-activated receptor-3 (PAR3), has multiple effects on many kinds of diseases [4]. F2RL2 modulates the polarized convergence between amyloid precursor protein (APP) and *β*-secretase *β*-site APP cleaving enzyme 1 (BACE1) in hippocampal neurons [5], as well as the planar cell polarity of inner ear hair cells [6]. Loss of F2RL2 enhances the prostatic tumorigenesis through promoting cell growth and facilitates lung adenocarcinoma metastasis by regulating 14-3-3zeta protein [7, 8]. Besides, F2RL2 expression is associated with the poor prognosis of ovarian cancer patients [9]. Recently, another protease-activated receptor, PAR2, is demonstrated to be able to protect against myocardial ischemia-reperfusion injury by regulating the lipoxygenase pathway and TRPV1 channels [10]. However, whether F2RL2 affects cardiovascular disease lacks supporting studies.

miR-582-5p has a strong inhibiting effect on various cancers. For instance, miR-582-5p suppresses the growth and invasion of osteosarcoma cells through targeting NOVA1 [11], inhibits bladder cancer-genesis through downregulating TTK expression [12], serves as an antioncogenic biomarker in acute myeloid leukemia (AML), hinders leukemia cell proliferation, induces cell apoptosis [13], and also hampers the migration and chemoresistant capabilities of colorectal cancer cells through targeting TNKS2 [14]. In addition, in hepatic ischemia/reperfusion injury, miR-582-5p is discovered to be lowly expressed [15]. In cerebral ischemic stroke, miR-582-5p overexpression is demonstrated to impede the neuronal cell apoptosis by regulating PAR1 [16]. Therefore, we speculated that miR-582-5p might also be involved in the process of MI. Previous researches verified that F2RL2 can be targeted by miR-429 and miR-483-3p in colorectal cancer and anaplastic thyroid cancer [17, 18]. Nevertheless, whether F2RL2 could be targeted by miR-582-5p is yet to be clarified.

Recently, there is a research discovering that miR-582-5p can be targeted by lncRNA nuclear paraspeckle assembly transcript 1 (NEAT1) to promote the epithelial-mesenchymal transition (EMT) of lung bronchial epithelial cells and the acquisition of cancer stem cell-like characteristics [19]. NEAT1 has the ability to enhance the myocardial ischemia-reperfusion injury by activating the MAPK signaling pathway [20]. However, whether NEAT1 impacts MI and targets miR-582-5p in MI needs to be expounded.

Therefore, in the present research, we established a MI mouse model and an oxygen-glucose deprivation- (OGD-) induced human cardiac myocyte (HCM) model and firstly investigated the role of the NEAT1/miR-582-5p/F2RL2 axis in MI.

## 2. Methods

### 2.1. Ethics Statement

A total of 60 8-week-old male C57BL/6 mice were purchased from ALF Biotechnology (Jiangsu, China). All animal experiments in the present research were authorized by the Committee of Experimental Animals of Nanfang Hospital (Z20210106X). All experiments involved in this study were performed at Guangdong.

### 2.2. MI Mouse Model Establishment

The 60 male C57BL/6 mice were distributed into 4 groups: the sham group (*n* = 15), the MI group (*n* = 15), the MI+shNC (*n* = 15), and the MI+shF2RL2 group (*n* = 15). The MI model was established by inducing MI in mice using ligation of the left anterior descending (LAD) coronary artery according to the previous research [2]. In brief, the mice were anesthetized with isoflurane (R510-22, RWD, Shenzhen, China). Then, their left ventricle was exposed through thoracotomy under sterile conditions. After that, the left coronary artery was ligated using a silk suture, and the incision was sutured. Mice in the sham group received all treatments as above-mentioned with the exception of ligation, while those in the other three groups received MI surgery. Twenty-four hours (h) after surgery, echocardiography was used to evaluate the left ventricular ejection fraction (EF). Mice with EF less than 20% or more than 50% were excluded for further study, and 2 mice were unselected (one in the MI group and the other in the MI+shF2RL2 group). Two weeks after MI surgery, lentiviruses for short hairpin RNA (shRNA) targeting F2RL2 (shF2RL2) were injected through the tail vein into the mice in the MI+shF2RL2 group, and shRNA negative control (shNC) lentiviruses were injected through the tail vein into the mice in the MI+shNC group. Two weeks later, the EF of all mice was evaluated again, and the mice were euthanized with isoflurane to collect myocardial tissues for further detection. Seven mice died during the model establishment (2 mice in the MI group, 3 mice in the MI+shNC group, and 2 mice in the MI+shF2RL2 group).

### 2.3. Echocardiographic Measurement

As previously described [1], transthoracic echocardiography was used to evaluate the EF of all mice using an ultrasound machine (Panoview *β*1500, Cold Spring Biotech, Taiwan, China). EF and fractional shortening (FS) were evaluated under M-model recoding with 30-MHz phased-array transducer.

### 2.4. Triphenyltetrazolium Chloride (TTC) and Evans Blue Staining

As previously delineated [1], the infarct area of experimental mice was detected using TTC and Evans blue staining. Briefly, after the mice were euthanatized with isoflurane, 2% Evans blue staining buffer (DK0051, Leagene Biotechnology, Beijing, China) was injected into the abdominal aorta and the hearts of all mice were harvested. The hearts were cut into 2 mm thick slices and further stained with 1% TTC buffer (DK0004, Leagene Biotechnology) at 37°C for 10 minutes (min). Finally, the infarct area was evaluated through Image-Pro Plus 5.0 software (Media Cybernetics, Wokingham, UK). The white area (unstained with either Evans blue or TTC) was the infarct area, the blue area was the noninfarct area, and the nonblue area (unstained with Evans blue) was the area at risk.

### 2.5. Hematoxylin-Eosin Staining

Pathological changes of mouse myocardial tissues were detected through hematoxylin-eosin staining using a staining kit (C0105M, Beyotime, Shanghai, China). In a nutshell, the myocardial tissues were collected and fixed with 4% paraformaldehyde buffer (DF0134, Leagene Biotechnology) for 24 h, after which the tissues were transparentized with xylene (B50009, Meryer, Shanghai, China), dehydrated with gradient alcohol, and further embedded into paraffin (A55701, OKA, Beijing, China). Then, the tissues were cut into 4 *μ*m slices which were dewaxed and stained with hematoxylin for 10 min, followed by being incubated with hydrochloric acid alcohol (C0163M, Beyotime) for 2 seconds (s). Then, the tissues were dyed with eosin for 1 min and incubated with xylene and neutral gum (IH0265, Leagene Biotechnology). Finally, the image of tissue was captured under a panoramic tissue microscopic imaging system (THUNDER; Leica, Wetzlar, Germany) at ×100 magnification.

### 2.6. Terminal Deoxynucleotidyl Transferase dUTP Nick-End Labeling (TUNEL) Staining

The cell apoptosis in mouse myocardial tissues was evaluated through TUNEL staining using a staining kit (C1088, Beyotime). In short, the prepared tissue slice was incubated with proteinase K (NH0058, Leagene Biotechnology) at 37°C for 20 min and then washed with PBS (IH0145, Leagene Biotechnology). Next, the slice was incubated with TUNEL working buffer at 37°C for 1 h in the dark and further stained with DAPI (DZ0125, Leagene Biotechnology). Ultimately, the tissue image was photographed using the DM2500 fluorescence microscope (Leica) under ×200 magnification.

### 2.7. Cell Culture

Human cardiac myocytes (HCMs; CP-H076) were purchased from Procell (Wuhan) and cultured in DMEM (11965092, Gibco, Waltham, Massachusetts, USA) blended with 10% FBS (10099141, Gibco) at 37°C with 5% CO_2_. To incubate OGD-modeled cells [21, 22], the HCMs were cultured in glucose-free DMEM (11966025, Gibco) with 10% FBS at 37°C with 93% N_2_, 2% O_2_, and 5% CO_2_ for 24 h and then collected for later assays.

### 2.8. Cell Transfection

Before transfection, shF2RL2, shRNA for lncRNA NEAT1 (shlncNEAT1), shNC, miR-582-5p mimic (M), mimic control (MC), miR-582-5p inhibitor (I), and inhibitor control (IC) were synthesized by RiboBio (Guangzhou, China). Then, the HCMs were cultured in 6-well plates until the cell confluence reached about 80%. Next, the above shRNA, miR-582-5p inhibitor, or miR-582-5p mimic was transfected into the cells using Lipofect transfection reagent (CZ0002, Leagene Biotechnology) for 48 h. Finally, the cells were collected for OGD treatment or further experiments.

### 2.9. Western Blot

The expression level of F2RL2 in myocardial tissues and HCMs was quantified using Western blot. The total protein was extracted from myocardial tissues and HCMs with RIPA lysis solution (PS0012, Leagene Biotechnology), followed by quantification using a BCA protein assay kit (PT0001, Leagene Biotechnology) under a microplate reader (Varioskan LUX, Thermo, Waltham, Massachusetts, USA) at a wavelength of 562 nm. Subsequently, the protein was denatured under 100°C after being mixed with loading buffer (PE0045, Leagene Biotechnology). 20 *μ*g protein was separated in SDS-PAGE (PE0018, Leagene Biotechnology) and transferred onto PVDF membrane (FFP24, Beyotime). Then, the membrane was blocked with nonfat milk for 2 h and incubated with primary antibodies against F2RL2 (ab40769, 150 kDa, 1: 500, Abcam, Cambridge, UK) and GAPDH (ab181602, 36 kDa, 1 : 5000, Abcam) at 4°C for 16 h. The next day, the secondary antibody goat-anti-rabbit IgG (ab205718, 1 : 10000, Abcam) was used to incubate the membrane for 2 h, followed by washing with TBST (PW0020, Leagene Biotechnology). After the membrane was covered with BeyoECL Moon buffer (P0018FS, Beyotime), the protein signaling in the membrane was detected under the ChemiDoc MP system (Bio-Rad, Hercules, California, USA).

### 2.10. EdU Staining

Cell proliferation was gauged using an EdU staining kit (KGA335-1000, KeyGEN BioTECH, Nanjing, China). In brief, after the HCMs were transfected and treated with OGD, the cells in 6-well plates were washed with PBS and incubated with EdU working buffer for 1 h. Then, the cells were fixed with 4% paraformaldehyde buffer for 20 min and incubated with glycine solution (R00325, Leagene Biotechnology) for 5 min. Following the washing with PBS and the incubation with 0.5% % Triton X-100 (R00280, Leagene Biotechnology) for 20 min, the cells were cultivated with Click-iT mixing solution for 30 min in the dark and then washed with PBS. Next, the cells were further incubated with DAPI for 20 min. Finally, the cell images were captured under a DM2500 fluorescence microscope at ×400 magnification.

### 2.11. Dual-Luciferase Reporter Assay

The binding sites predicted by StarBase between NEAT1 with miR-582-5p and between miR-582-5p with F2RL2 were further verified by dual-luciferase reporter assay. In a word, wide-type (WT) sequence of NEAT1 containing miR-582-5p binding sites (NEAT1-WT) and the corresponding mutant (MUT) sequence (NEAT1-MUT) were cloned into pGL3-Basic vector (VT1554, YouBio, Hunan, China), respectively. Besides, the WT sequence of F2RL2 incorporating miR-582-5p binding sites (F2RL2-WT) and the corresponding F2RL2-MUT sequence were also cloned into pGL3-Basic vector, respectively. Thereafter, the above vectors were transfected into the HCMs together with miR-582-5p mimic and mimic control for 48 h. Subsequently, the HCMs were collected and treated using Dual-Luciferase Reporter Assay System Kit (E1960, Promega, Shanghai, China). At last, the cell relative luciferase activity was determined using a SpectraMax reader (Molecular Devices, Shanghai, China).

### 2.12. Real-Time Quantitative Reverse Transcription-PCR (qRT-PCR)

The expressions of NEAT1, F2RL2, and miR-582-5p in mouse myocardial tissues and HCMs were detected using qRT-PCR. Briefly, total RNA and miRNA in the samples were extracted using total RNA extraction reagent (10606ES60, YEASEN, Shanghai, China) and an miRNA kit (19331ES08, YEASEN), respectively. Then, the RNA concentration was quantified with a UV spectrophotometer (NanoDrop, Thermo). The RNA was reverse-transcribed into cDNA by a PrimeScript RT reagent kit (RR047A, TaKaRa, Tokyo, Japan). The cDNA was mixed with SYBR green master buffer (11203ES03, YEASEN) and gene primers, and the above mixture was placed into 7500 Instrument (Applied Biosystems, Waltham, Massachusetts, USA) to perform an amplification reaction. The reaction condition was set as follows: predenaturing at 95°C for 5 min, denaturing at 95°C for 10 s, annealing at 55°C for 20 s, and extension at 72°C for 30 s, with 40 cycles of the process from denaturing to extension. Primers are displayed in Table 1.

### 2.13. Cell Counting Kit-8 (CCK-8) Assay

When the HCMs were transfected, treated with OGD and cultured for 24 h and 48 h, the cell viability was evaluated using CCK-8 assay. All in all, after treatment, the HCMs were collected and placed into a 96-well plate at a density of 1,000 cells/well. Following the 24 h and 48 h of normal culture of HCMs, 10 *μ*l CCK-8 solution (KGA317, KeyGEN BioTECH) was added into each well to incubate the cells for 3 h. Finally, the optical density in cells of each well was determined using a microplate reader at a wavelength of 450 nm.

### 2.14. Cell Apoptosis Assay

After the HCMs were transfected and subjected to OGD, the cell apoptosis was evaluated through flow cytometry using an Annexin V-FITC/Propidium Iodide (PI) kit (KGA108, KeyGEN BioTECH). Briefly speaking, the treated 1 × 10^5^ HCMs were collected and resuspended with 500 *μ*l binding buffer, after which 5 *μ*l Annexin V-FITC and 5 *μ*l PI were added for 15 min of incubation in the dark. Finally, the apoptosis signal in cells was detected with the assistance of an Attune NxT flow cytometer (Thermo).

### 2.15. Statistical Analysis

Data from two groups and from multiple groups were analyzed using *T*-test and one-way ANOVA, respectively. All data analyses were implemented using GraphPad 8.0 software. Statistical data were exhibited as mean ± standard deviation (SD). *P* < 0.05 was defined as statistical significance.

## 3. Results

### 3.1. NEAT1 and F2RL2 Were Highly Expressed while miR-582-5p Was Lowly Expressed in MI Mice

**Figure 1:**
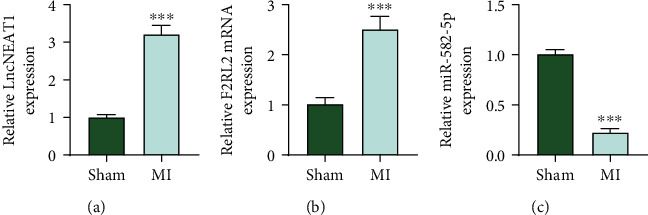
NEAT1 and F2RL2 were highly expressed while miR-582-5p was lowly expressed in MI mice. (a–c) The expressions of NEAT1, F2RL2, and miR-582-5p in myocardial tissues of MI mice were examined by qRT-PCR. (^∗∗∗^*P* < 0.001, vs. sham) (NEAT1: nuclear paraspeckle assembly transcript 1; F2RL2: coagulation factor II thrombin receptor like 2; qRT-PCR: quantitative RT-PCR; MI: myocardial infarction).

At the time that the MI mouse model was established, the expressions of NEAT1, F2RL2, and miR-582-5p were firstly examined. As illustrated in Figures 1(a)–1(c), NEAT1 (Figure 1(a)) and F2RL2 (Figure 1(b)) levels were upregulated (*P* < 0.001), while miR-582-5p (Figure 1(c)) level was downregulated in myocardial tissues of MI mice (*P* < 0.001).

### 3.2. F2RL2 Downregulation Ameliorated Left Ventricular Function, Infarct Area, and Cell Apoptosis in Myocardial Tissues of MI Mice

**Figure 2:**
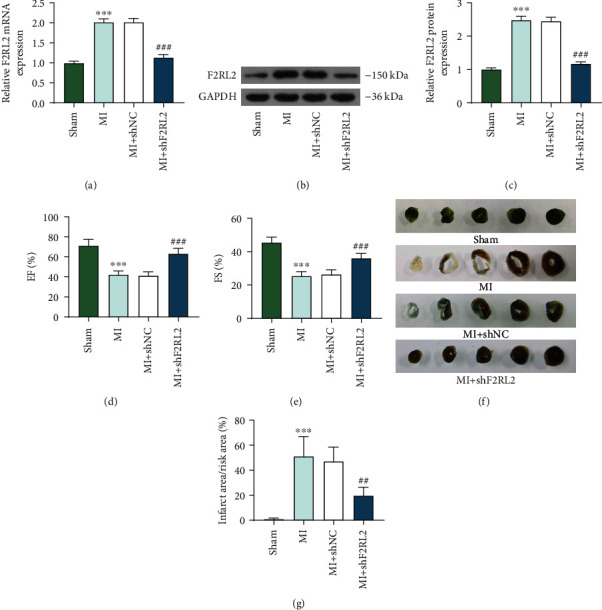
F2RL2 downregulation ameliorated left ventricular function and infarct area of MI mice. (a–c) The expression of F2RL2 in myocardial tissues of MI mice was examined by qRT-PCR and Western blot. (d, e) The EF (d) and FS (e) of MI mice were evaluated using echocardiography. (f, g) The infarct area of mice was examined by TTC and Evans blue staining assays. (^∗∗∗^*P* < 0.001, vs. sham; ^##^*P* < 0.01 and ^###^*P* < 0.001, vs. MI+shNC) (F2RL2: coagulation factor II thrombin receptor like 2; MI: myocardial infarction; TTC: triphenyltetrazolium chloride; EF: ejection fraction; SF: fractional shortening).

**Figure 3:**
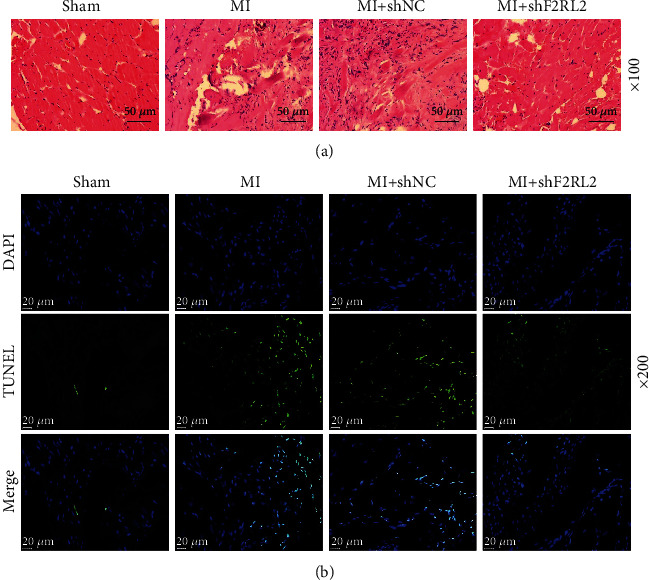
F2RL2 downregulation mitigated the cell apoptosis in myocardial tissues of MI mice. (a) The pathological changes of mouse myocardial tissues were detected using hematoxylin-eosin staining (magnification ×100). (b) The cell apoptosis in myocardial tissues was evaluated using TUNEL staining (magnification ×200). (F2RL2: coagulation factor II thrombin receptor like 2; MI: myocardial infarction).

To verify the role of F2RL2 in MI mice, lentiviruses for shF2RL2 were injected into the MI mice. Firstly, the mRNA and protein expressions of F2RL2 were detected, which were found to be increased in MI mice, while shF2RL2 inhibited the expression of F2RL2 in MI mice (*P* < 0.01, Figures 2(a)–2(c)). In addition, the evaluation of left ventricular function revealed that the EF (Figure 2(d)) and FS (Figure 2(e)) of MI mice were reduced (*P* < 0.001), while shF2RL2 elevated the EF and FS of MI mice (*P* < 0.001). Then, the infarct area of mice was assessed and the representative images were exhibited in Figure 2(f). The data after analyses indicated (Figure 2(g)) that MI mice had a larger infarct area (*P* < 0.001) which was lessened by shF2RL2 (*P* < 0.01). Next, the pathological changes of mouse myocardial tissues were detected using hematoxylin-eosin staining. According to Figure 3(a), in the sham group, the myocardial tissue was normal; in the MI and MI+shNC groups, the cardiomyocytes were disorderly arranged and loosely connected, with increased interstitial inflammatory exudation; and in the MI+shF2RL2 group, the cardiomyocytes were arranged neatly and the interstitial inflammatory exudation was reduced when compared with those in the MI+shNC group. In addition, the cell apoptosis in myocardial tissues was evaluated using TUNEL staining. It could be noted from Figure 3(b) that the number of TUNEL-positive cells was higher in the MI and MI+shNC groups than in the sham group, while the number was lower in the MI+shF2RL2 group than in the MI+shNC group. The above discoveries demonstrated that F2RL2 downregulation ameliorated left ventricular function, infarct area, and cell apoptosis in myocardial tissues of MI mice.

### 3.3. F2RL2 Expression Was Upregulated in OGD-Induced HCMs

**Figure 4:**
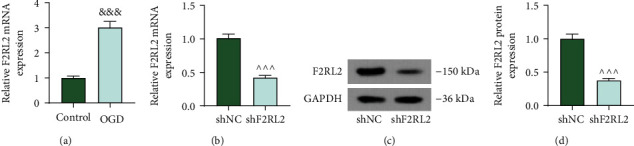
F2RL2 expression was upregulated in OGD-induced HCMs. (a) The expression of F2RL2 in OGD-induced HCMs was evaluated using qRT-PCR. (b–d) After cells were transfected with shF2RL2, the expression of F2RL2 in HCMs was examined using qRT-PCR (b) and Western blot (c, d). (^&&&^*P* < 0.001, vs. control; ^^^^^*P* < 0.001, vs. shNC) (HCMs: human cardiac myocytes; F2RL2: coagulation factor II thrombin receptor like 2; OGD: oxygen-glucose deprivation; qRT-PCR: quantitative RT-PCR).

To further verify the role of F2RL2 in MI *in vitro*, the OGD-induced HCM model was established to mimic the MI *in vitro*. The expression of F2RL2 was discovered to be upregulated in OGD-induced HCMs (*P* < 0.001; Figure 4(a)). Therefore, we silenced F2RL2 expression in HCMs through transfection with shF2RL2 (*P* < 0.001; Figures 4(b)–4(d)) for follow-up experiments.

### 3.4. F2RL2 Downregulation Ameliorated the Inhibited Viability and Proliferation and the Promoted Apoptosis of OGD-Induced HCMs

**Figure 5:**
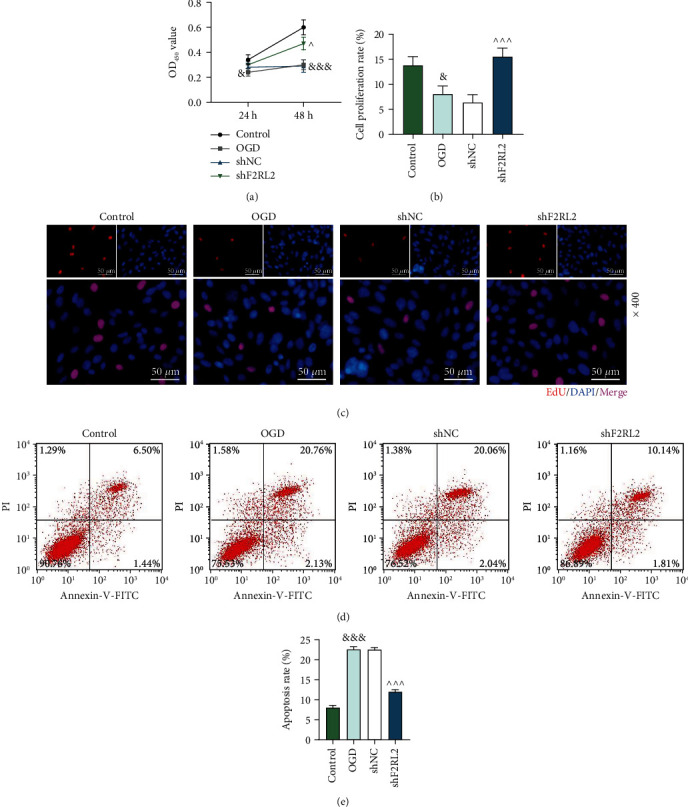
F2RL2 downregulation ameliorated the decreased viability and proliferation and the increased apoptosis of OGD-induced HCMs. (a) After cells received transfection and OGD induction, the viability of HCMs was detected by CCK-8 assay. (b, c) After cells received transfection and OGD induction, the proliferation of HCMs was evaluated using EdU staining (magnification ×400). (d, e) After cells received transfection and OGD induction, the apoptosis of HCMs was examined using flow cytometry. (^&^*P* < 0.05 and ^&&&^*P* < 0.001, vs. control; ^^^*P* < 0.05 and ^^^^^*P* < 0.001, vs. shNC) (HCMs: human cardiac myocytes; F2RL2: coagulation factor II thrombin receptor like 2; OGD: oxygen-glucose deprivation).

After F2RL2 expression was downregulated in HCMs, the effect of F2RL2 on OGD-induced cells was explored. The data of CCK-8 assay confirmed that OGD reduced the OD value of HCMs both at 24 h and 48 h (*P* < 0.05, Figure 5(a)), while shF2RL2 elevated the OD value at 48 h in OGD-induced HCMs (*P* < 0.001, Figure 5(a)). What is more, cell proliferation was evaluated using EdU staining, with the results verifying that the proliferation ability of HCMs was hindered by OGD (*P* < 0.05, Figures 5(b) and 5(c)), but shF2RL2 neutralized the effect of OGD and boosted the proliferation (*P* < 0.001, Figures 5(b) and 5(c)). Besides, the apoptosis of OGD-induced HCMs was determined by flow cytometry, as illustrated in Figures 5(d) and 5(e) that the apoptosis ability of HCMs was first enhanced by OGD (*P* < 0.001) but then inhibited with the further transfection of shF2RL2 (*P* < 0.001). These phenomena proved that F2RL2 downregulation ameliorated the decreased viability and proliferation and the increased apoptosis of OGD-induced HCMs.

### 3.5. F2RL2 Was Targeted by miR-582-5p Which Was Sponged by NEAT1 in HCMs

**Figure 6:**
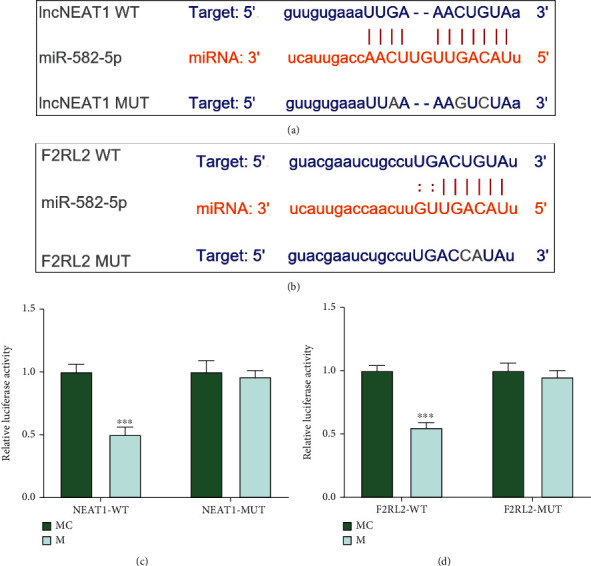
F2RL2 was targeted by miR-582-5p which was sponged by NEAT1 in HCMs. (a, b) The binding sites between NEAT1 with miR-582-5p (a) and between F2RL2 with miR-582-5p (b) were predicted through StarBase. (c, d) The targeting relationships between NEAT1 with miR-582-5p (c) and between F2RL2 with miR-582-5p (d) in HCMs were verified by dual-luciferase reporter assay. (^∗∗∗^*P* < 0.001, vs. MC) (HCMs: human cardiac myocytes; F2RL2: coagulation factor II thrombin receptor like 2; NEAT1: nuclear paraspeckle assembly transcript 1).

The upstream regulatory genes of F2RL2 were explored. Through prediction in StarBase, binding sites between NEAT1 with miR-582-5p (Figure 6(a)) and between F2RL2 with miR-582-5p (Figure 6(b)) were found out. Therefore, dual-luciferase reporter assay was performed, as shown in Figures 6(c) and 6(d) that cell luciferase activity was weakened after cells were transfected with miR-582-5p mimic containing NEAT1-WT (*P* < 0.001; Figure 6(c)) and F2RL2-WT (*P* < 0.001; Figure 6(d)), while no changes of cell luciferase activity were discovered in cells after being transfected with miR-582-5p mimic encompassing NEAT1-MUT (Figure 6(c)) and F2RL2-MUT (Figure 6(d)). These indicated that F2RL2 was targeted by miR-582-5p which was sponged by NEAT1 in HCMs.

### 3.6. miR-582-5p Inhibitor Suppressed the Viability and Enhanced the Apoptosis of OGD-Induced HCMs by Upregulating F2RL2 Expression

**Figure 7:**
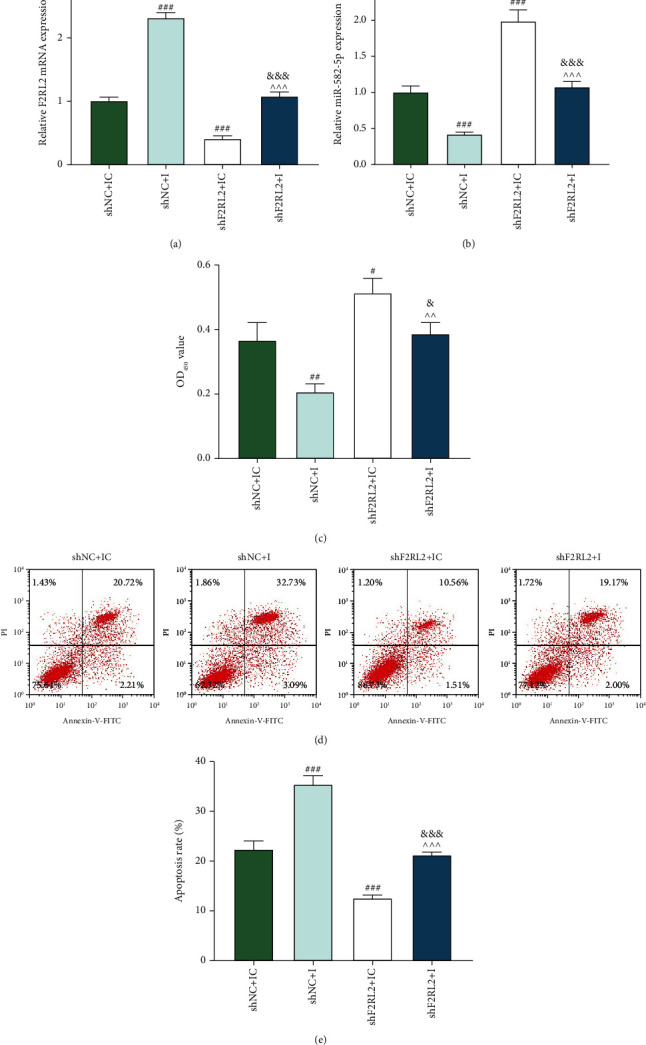
miR-582-5p inhibitor suppressed the viability and enhanced the apoptosis of OGD-induced HCMs by upregulating F2RL2 expression. (a, b) After cells received transfection and OGD induction, the expressions of F2RL2 (a) and miR-582-5p (b) in HCMs were evaluated by qRT-PCR. (c) After cells received transfection and OGD induction, the viability of HCMs was measured by CCK-8 assay. (d, e) After cells received transfection and OGD induction, the apoptosis of HCMs was determined by flow cytometry. (^#^*P* < 0.05, ^##^*P* < 0.01, and ^###^*P* < 0.001, vs. shNC+IC; ^^^^^*P* < 0.001, vs. shNC+I; ^&^*P* < 0.05 and ^&&&^*P* < 0.001, vs. shF2RL2+IC) (HCMs: human cardiac myocytes; F2RL2: coagulation factor II thrombin receptor like 2; OGD: oxygen-glucose deprivation).

The expressions of F2RL2 and miR-582-5p in OGD-induced HCMs after transfection were evaluated. According to Figures 7(a) and 7(b), miR-582-5p inhibitor upregulated F2RL2 expression and downregulated miR-582-5p expression (*P* < 0.001), while shF2RL2 produced the inverse effects on these two expressions (*P* < 0.001). Furthermore, miR-582-5p inhibitor and shF2RL2 mutually reversed their roles in F2RL2 and miR-582-5p expressions (*P* < 0.001). Then, the viability (Figure 7(c)) and apoptosis (Figures 7(d) and 7(e)) of OGD-induced HCMs were evaluated, revealing that miR-582-5p inhibitor suppressed the viability (*P* < 0.01) and enhanced the apoptosis (*P* < 0.001) of OGD-induced HCMs, whereas shF2RL2 facilitated the viability (*P* < 0.05) and blocked the apoptosis (*P* < 0.001). Furthermore, miR-582-5p inhibitor and shF2RL2 mutually affected their roles in cell viability and apoptosis (*P* < 0.05). These results demonstrated that miR-582-5p inhibitor suppressed the viability and enhanced the apoptosis of OGD-induced HCMs by upregulating F2RL2 expression.

### 3.7. NEAT1 Downregulation Facilitated the Viability and Impeded the Apoptosis of OGD-Induced HCMs by Upregulating miR-582-5p Expression

**Figure 8:**
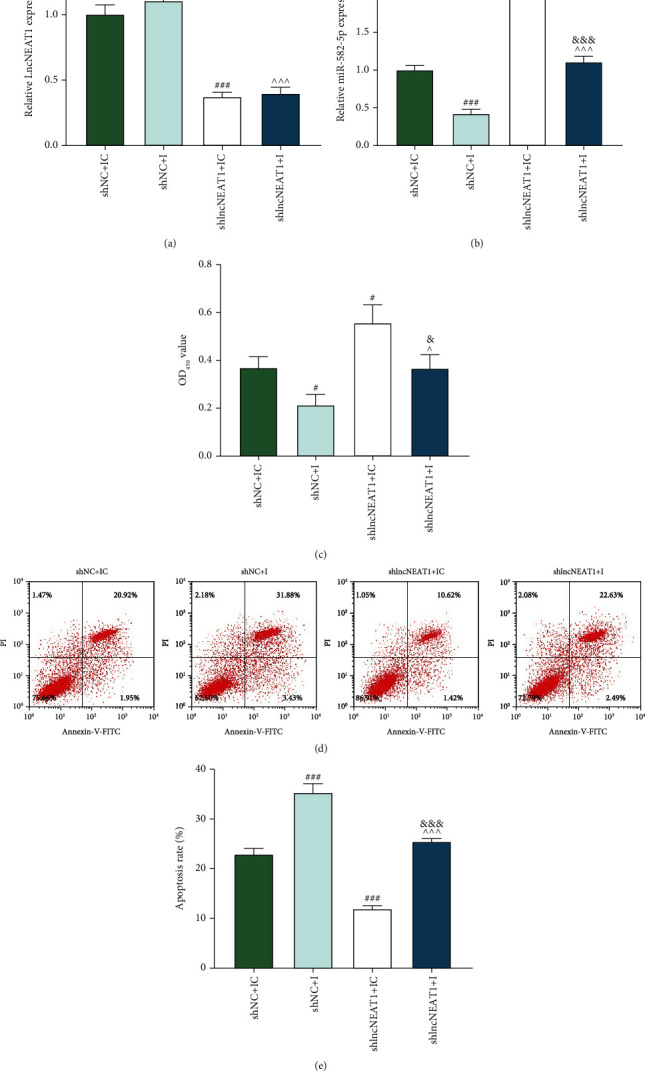
NEAT1 downregulation promoted the viability and suppressed the apoptosis of OGD-induced HCMs by upregulating miR-582-5p expression. (a, b) After cells received transfection and OGD induction, the expressions of NEAT1 (a) and miR-582-5p (b) in HCMs were evaluated by qRT-PCR. (c) After cells received transfection and OGD induction, the viability of HCMs was detected by CCK-8 assay. (d, e) After cells received transfection and OGD induction, the apoptosis of HCMs was evaluated by flow cytometry. (^#^*P* < 0.05 and ^###^*P*<0.001, vs. shNC+IC; ^^^*P* < 0.05 and ^^^^^*P* < 0.001, vs. shNC+I; ^&^*P* < 0.05 and ^&&&^*P* < 0.001, vs. shlncNEAT1+IC) (HCMs: human cardiac myocytes; NEAT1: nuclear paraspeckle assembly transcript 1; OGD: oxygen-glucose deprivation).

The expressions of NEAT1 and miR-582-5p in OGD-induced HCMs after transfection were evaluated. As displayed in Figures 8(a) and 8(b), miR-582-5p inhibitor resulted in downregulated miR-582-5p (*P* < 0.001), yet NEAT1 silencing brought about downregulated NEAT1 and upregulated miR-582-5p (*P* < 0.001). Besides, the promoting effect of NEAT1 silencing on miR-582-5p level was offset by miR-582-5p inhibitor (*P* < 0.001). Then, the viability (Figure 8(c)) and apoptosis (Figures 8(d) and 8(e)) of OGD-induced HCMs were evaluated. The discoveries disclosed that miR-582-5p inhibitor suppressed the viability (*P* < 0.05) and furthered the apoptosis (*P* < 0.001) of OGD-induced HCMs, whereas the effects of NEAT1 silencing were opposite to those of miR-582-5p inhibitor. Moreover, miR-582-5p inhibitor and NEAT1 silencing mutually impacted their roles in cell viability and apoptosis (*P* < 0.05). These findings demonstrated that NEAT1 downregulation boosted the viability and impeded the apoptosis of OGD-induced HCMs by upregulating miR-582-5p expression.

## 4. Discussion

F2RL2 has been proved to have multiple effects on different types of diseases, such as regulating the planar cell polarity of inner ear hair cells, epithelial cells, and hippocampal neurons [5, 6, 23], and play a role in modulating the tumorigenesis of skin cancer, colorectal cancer, ovarian cancer, lung cancer, thyroid cancer, prostatic cancer, and so on [7–9, 17, 18, 24]. However, researches pertaining to the role of F2RL2 in MI are lacking as far as we are concerned. Recently, PAR2, which is also a protease-activated receptor, is confirmed to protect against myocardial ischemia-reperfusion injury by regulating lipoxygenase pathway and TRPV1 channels [10]. We then wondered if F2RL2 makes an impact upon MI. After we established the MI mouse model, F2RL2 expression was discovered to be upregulated in MI mouse myocardial tissues. Besides, F2RL2 downregulation mitigated the left ventricular function and infarct area of MI mice, denoting that F2RL2 downregulation had an improving effect on MI. It is well known that apoptosis leads to cell loss following myocardial infarction, which takes part in the pathogenesis of myocardial injury during myocardial infarction [25]. In this research, we further uncovered that F2RL2 downregulation retarded cell apoptosis in myocardial tissue of MI mice. All these phenomena corroborated that F2RL2 was highly expressed in MI and its downregulation had an inhibitory effect on MI.

Previous studies have reported that F2RL2 can be targeted by miR-429 and miR-483-3p in colorectal cancer and anaplastic thyroid cancer, respectively [17, 18]. In this study, it was the first time to unveil that miR-582-5p had binding sites with F2RL2. miR-582-5p is widely proved to have a suppressing effect on tumors such as osteosarcoma, bladder cancer, AML, and colorectal cancer [11–14]. Besides, the low expression level of miR-582-5p appeared in hepatic ischemia/reperfusion injury [15]. Herein, we discovered that miR-582-5p expression was downregulated in MI mouse myocardial tissues, indicating that miR-582-5p might have a regulatory effect on MI. In cerebral ischemic stroke, miR-582-5p overexpression is demonstrated to hamper neuronal cell apoptosis by modulating PAR1 which is also a protease-activated receptor [16]. In our present research, we affirmed that miR-582-5p targeted F2RL2 and negatively regulated the expression of F2RL2. To further verify the effect of the miR-582-5p/F2RL2 axis on MI, OGD-induced HCMs were transfected with miR-582-5p inhibitor and shF2RL2. miR-582-5p inhibitor was revealed to reduce the viability while inducing apoptosis of OGD-induced HCMs. F2RL2 downregulation not only had opposite effects but also reversed the role of miR-582-5p inhibitor in OGD-induced HCMs, signifying that miR-582-5p downregulation positively affected MI by increasing the expression of F2RL2.

Furthermore, lncRNA which could regulate the miR-582-5p/F2RL2 axis in MI was also explored in this study. After bioinformatics analysis and experiment verification, NEAT1 was proved to target miR-582-5p and negatively modulate miR-582-5p expression, which was similar to a previous research uncovering that miR-582-5p can be targeted by NEAT1 to promote the EMT of lung bronchial epithelial cells and the acquisition of cancer stem cell-like characteristics [19]. NEAT1 is widely researched in different kinds of diseases and possesses proinflammatory, profibrosis, and procancer effects [26, 27, 28]. Additionally, in studies involving cardiovascular disease, Wang et al. clarified that NEAT1 knockdown can improve ischemia/reperfusion-induced cardiac insufficiency in rats and LPS-induced myocardial injury in mice by inhibiting the TLR2/NF-*κ*B signaling pathway [29]; and Du et al. demonstrated that NEAT1 aggravates the myocardial ischemia-reperfusion injury in mice by activating the MAPK signaling pathway [20]. Consistent with the above literature, a high level of NEAT1 was found in MI mouse myocardial tissues, and NEAT1 knockdown reinforced the viability and blocked the apoptosis of OGD-induced HCMs, the tendencies of which were further neutralized by miR-582-5p downregulation. These pieces of evidence suggested that NEAT1 knockdown improved MI by regulating the miR-582-5p/F2RL2 axis.

## 5. Conclusion

In a word, our research authenticates that NEAT1 and F2RL2 expressions are upregulated and miR-582-5p expression is downregulated in MI mice. NEAT1 downregulation ameliorates MI by modulating the miR-582-5p/F2RL2 axis, thus providing ideal biomarkers available to prevent, diagnose, and treat MI.

## Figures and Tables

**Table 1:** All primers used in qRT-PCR in this study.

Target gene	Forward primers, 5′-3′	Reverse primers, 5′-3′
mmu-NEAT1	TGCTGCCTTTTCTGTTCCTT	CTAGCTAGCTTTGGGTAGGGAAGT
mmu-F2RL2	GCCAGTCACTGTTTGCCAAAG	CCAGCCCTCTATGTCAGAAAGT
mmu-miR-582-5p	ACGTCGTATCCAGTGCAATTG	GTCGTATCCAGTGCGTGTCG
hsa-NEAT1	CTTCCTCCCTTTAACTTATCCATTCAC	CTCTTCCTCCACCATTACCAACAATAC
hsa-F2RL2	GCAAAGCCAACCTTACCCATT	GAGGTAGATGGCAGGTATCAGT
hsa-miR-582-5p	CTGTCGTATCCAGTGCAATTGC	GTCGTATCCAGTGCGTGTCG
mmu-GAPDH	AGGTCGGTGTGAACGGATTTG	GGGGTCGTTGATGGCAACA
hsa-GAPDH	GGAGCGAGATCCCTCCAAAAT	GGCTGTTGTCATACTTCTCATGG
mmu-U6	CTGGTAGGGTGCTCGCTTCGGCAG	CAACTGGTGTCGTGGAGTCGGC
hsa-U6	CTCGCTTCGGCAGCACA	AACGCTTCACGAATTTGCGT

## Data Availability

The analyzed data sets generated during the study are available from the corresponding author on reasonable request.
